# The role of IL-18 in addition to Th17 cytokines in rheumatoid arthritis development and treatment in women

**DOI:** 10.1038/s41598-021-94841-x

**Published:** 2021-07-28

**Authors:** Georgi Vasilev, Irena Manolova, Mariana Ivanova, Iskren Stanilov, Lyuba Miteva, Spaska Stanilova

**Affiliations:** 1 Laboratory of Clinical Immunology, University Hospital “St. Iv. Rilski”, Medical University of Sofia, Sofia, Bulgaria; 2 Department of Molecular Biology, Immunology and Medical Genetics, Medical Faculty, Trakia University, Armeiska 11 Str, Stara Zagora, 6000 Bulgaria; 3 Clinic of Rheumatology, University Hospital “St. Iv. Rilski”, Medical University of Sofia, Sofia, Bulgaria

**Keywords:** Autoimmunity, Cytokines, Immunological disorders, Immunology

## Abstract

We aimed to analyze serum pro-inflammatory profiles of female rheumatoid arthritis (RA) patients and compare them with healthy women to establish the relative importance of pro-inflammatory cytokines in RA and their relation with different treatment regimens. Levels of six cytokines were determined by ELISA assays. A supervised dimensionality reducing approach (PLS-DA Analysis) was applied. All of the cytokines assayed were significantly elevated in the sera of RA female patients than healthy controls with fold change: 21-fold for IL-6; 6.1-fold for IL-17A; 2.5-fold for IL-23; 2.3-fold for IL-18; 1.94-fold for TNF-α; 1.7-fold for IL-12p40. According to the results of the PLS-DA analysis, IL-17A, IL-18, and TNF-α were of higher importance rank compared to IL-23 and IL-12p40. Women in the early stage of RA displayed significantly elevated IL-17A levels than those with longer disease duration: 8.04 pg/ml [8.04–175.3] vs 4.64 pg/ml [2.95–13.31], *p* = 0.007. IL-6 serum levels were related to higher disease activity. We have demonstrated altered cytokine production within female RA patients on different treatment regimens. Those on Tocilizumab therapy showed elevated IL-6 levels and decreased IL-17A versus the rest of the patients’ subgroups. In conclusion, our data support the pivotal role of IL-18 in addition to IL-6, IL-17A, and TNF-α as the hierarchical cytokines in the pathogenesis of RA, particularly valid for women. Therapy with biological agents targeting IL-18 in addition to the Th17 axis may be an adequate approach in RA patients.

## Introduction

Rheumatoid arthritis (RA) is a debilitating chronic progressive autoimmune disorder that displays relatively constant prevalence across different populations, strong female preponderance, and affects up to 1% of the population worldwide^1,2^. Although RA represents a heterogeneous phenotype, its most distinctive features are chronic synovitis and hyperplasia, bone erosion, cartilage destruction, autoantibody production, and systemic manifestations outside the joints as well^3^. Immunopathogenetically, the chain of events leading to RA development could be linked to loss of peripheral self-tolerance, activation and survival of autoreactive T and B lymphocytes, and over-activation of pro-inflammatory cytokines pathways^4^. In case of inadequate or no treatment, chronic inflammatory synovitis may progress to irreversible joint damage, loss of function, and even physical handicap, thus bringing a substantial burden on individuals and society^5^. Although the recent breakthroughs in RA treatment and the advent of novel therapeutic avenues such as biological disease-modifying anti-rheumatic drugs (bDMARDs) and conventional synthetic DMARDs (csDMARDs), many unmet demands remain^6,7^. Considering recent data in the literature, 30% of RA patients remain refractory to anti-TNF-α signaling inhibitors, and only a few could sustain persistent and longstanding remission^6,8^. Moreover, the benefit from bDMARDs and csDMARDs is often accompanied by severe adverse effects such as the increased risk of infections, tuberculosis, malignancies, thrombosis, granulocytopenia^9,10^. This further emphasizes the need for the development of novel pharmaceutical avenues and curative strategies for RA.

In this line of thought, the concept of the “disease taxonomy based on the hierarchical function of pro-inflammatory cytokine pathways” has drawn considerable interest. It postulates the existence of a shared and highly conserved framework of dysregulations in cytokine signaling pathways central to particular autoimmune diseases^11^. These cytokine pathways were termed as “vulnerable nodes”, and so far, TNF-α, IL-17, and IL-6 were identified as such “vulnerable” targets in RA^11^. Moreover, the idea of vulnerable nodes suggests that novel treatment protocols should be even more centered on inhibition of such weak points, thus offering specific RA “tailored” therapy that better suits RA patients’ demands. In this regard, we focused our cross-sectional study on this concept. We aimed to investigate and compare circulating cytokine levels across healthy controls and female RA patients. To identify such vulnerable nodes, we applied a supervised dimensionality reducing approach to examine the contribution or importance of studied cytokines for distinguishing between healthy women and female RA patients. Furthermore, we investigated differences in cytokine levels within female RA patients according to the severity and different treatment regimens.

## Results

### Comparison of the serum cytokine levels between female RA patients and healthy women.

The concentrations of cytokines assayed in serum samples of RA female patients and healthy women are shown in Table 1.

**Table 1:** Serum cytokines in RA female patients and healthy women.

Cytokine levels (pg/ml)	RA female patients					Healthy women				
	Mean ± SD	Q1 (p25)	Q2 (Median)	Q3 (p75)	(Range)	Mean ± SD	Q1 (p25)	Q2 (Median)	Q3 (p75)	Range
IL-6	26.23 ± 43.7	2.85	10.01	36.39	(0.0–64.87)	1.25 ± 1.4	0.20	0.70	2.0	0.0–5.0
IL-17A	15.52 ± 31.27	2.95	5.22	14.38	1.31–208.7	2.6 ± 2.0	1.61	2.26	3.41	0.0–9.77
IL-18	296.01 ± 187.5	175.5	265.0	365.0	35.64–977.0	128.76 ± 72.1	99.2	120.2	164.8	29.2–386.3
TNF-α	7.11 ± 7.9	2.92	5.37	8.63	0.52–62.42	3.7 ± 2.3	1.4	3.8	5.4	0.84–8.7
IL-23	45.27 ± 87.9	8.51	14.33	24.8	0.0–471.74	16.2 ± 14.0	6.9	11.1	25.7	0.0–52.33
IL-12p40	161.76 ± 118.2	73.62	124.82	235.4	7.88–532.5	94.5 ± 73.7	46.2	69.7	116.3	6.8–399.65

TNF-α, IL-12p40, IL-17A, and IL-18 were detectable in all RA patients, whereas detectable circulating levels of IL-6 and IL-23 were present in 71 (91%), 76 (97%), and 74 (95%) of RA patients, respectively. In the control cohort, IL-6, IL-17A, and IL-23 were detected in 39 (76%), 46 (90%), and 47 (92%) of healthy women, respectively. All of the healthy controls showed detectable levels of TNF-α, IL-12p40, and IL-18.

**Figure 1:**
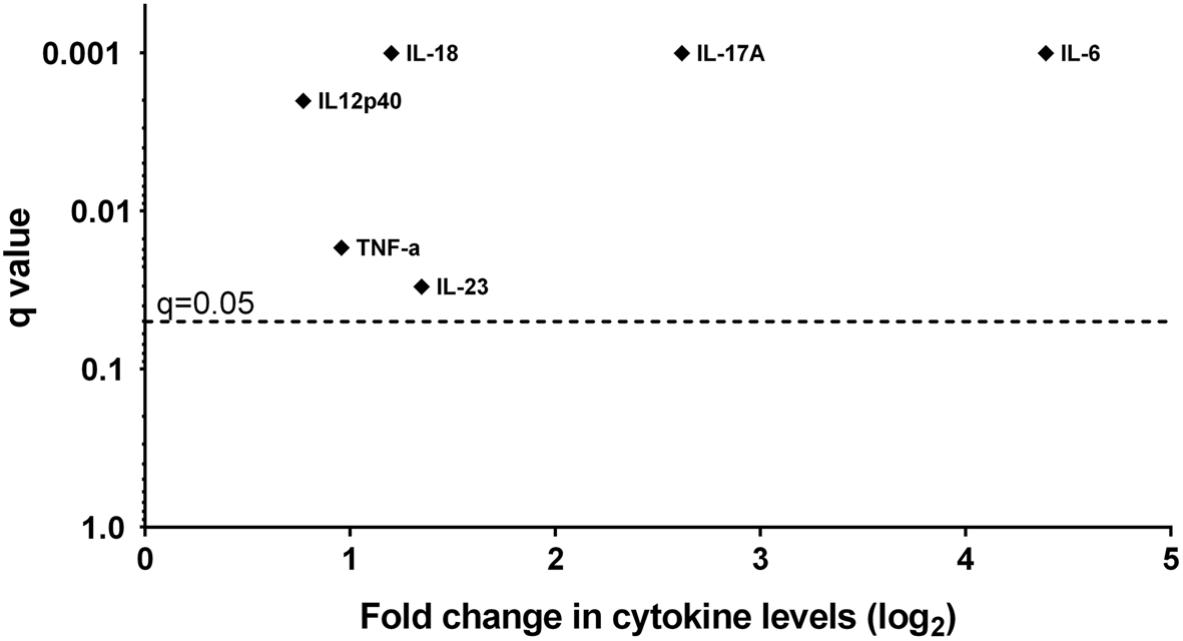
Volcano plot displaying the fold change (log_2_) in tested cytokine levels in sera of female patients with RA as opposed to healthy females.

The serum levels of all cytokines in RA patients showed a skewed distribution, which was expected due to the RA cohort’s heterogeneity assayed. In healthy individuals, IL12p40 and IL-18 also showed wide variations. All of the cytokines assayed were significantly elevated in the sera of RA patients compared to healthy controls with the dominance of IL-6 and IL-17A over the others: IL-6 (q = 0.001); IL-17A (q = 0.001); TNF-α (q = 0.017); IL-23 (q = 0.03.); IL-18 (q = 0.001.); IL-12p40 (q = 0.002), (corrections due to multiple testing were applied under Benjamini and Hochberg method; False Detective Rate was set at 5%). For greater demonstrativeness, the fold change, expressed as log_2_ in tested cytokine levels in sera of female patients with RA as opposed to healthy females, is presented in Fig. 1 (21 fold for IL-6; 6.1 fold for IL-17A; 2.5 fold for IL-23; 2.3 fold for IL-18; 1.94 fold for TNF-α; 1.7 fold for IL12p40).

In RA patients, weakly positive correlation was observed between IL-6 and IL-12p40 (Spearman’s R = 0.3, *p* = 0.036), as well for TNF-α with IL-18 (Spearman’s R = 0.3, *p* = 0.016).

### Partial least squares-discriminant analysis

**Figure 2:**
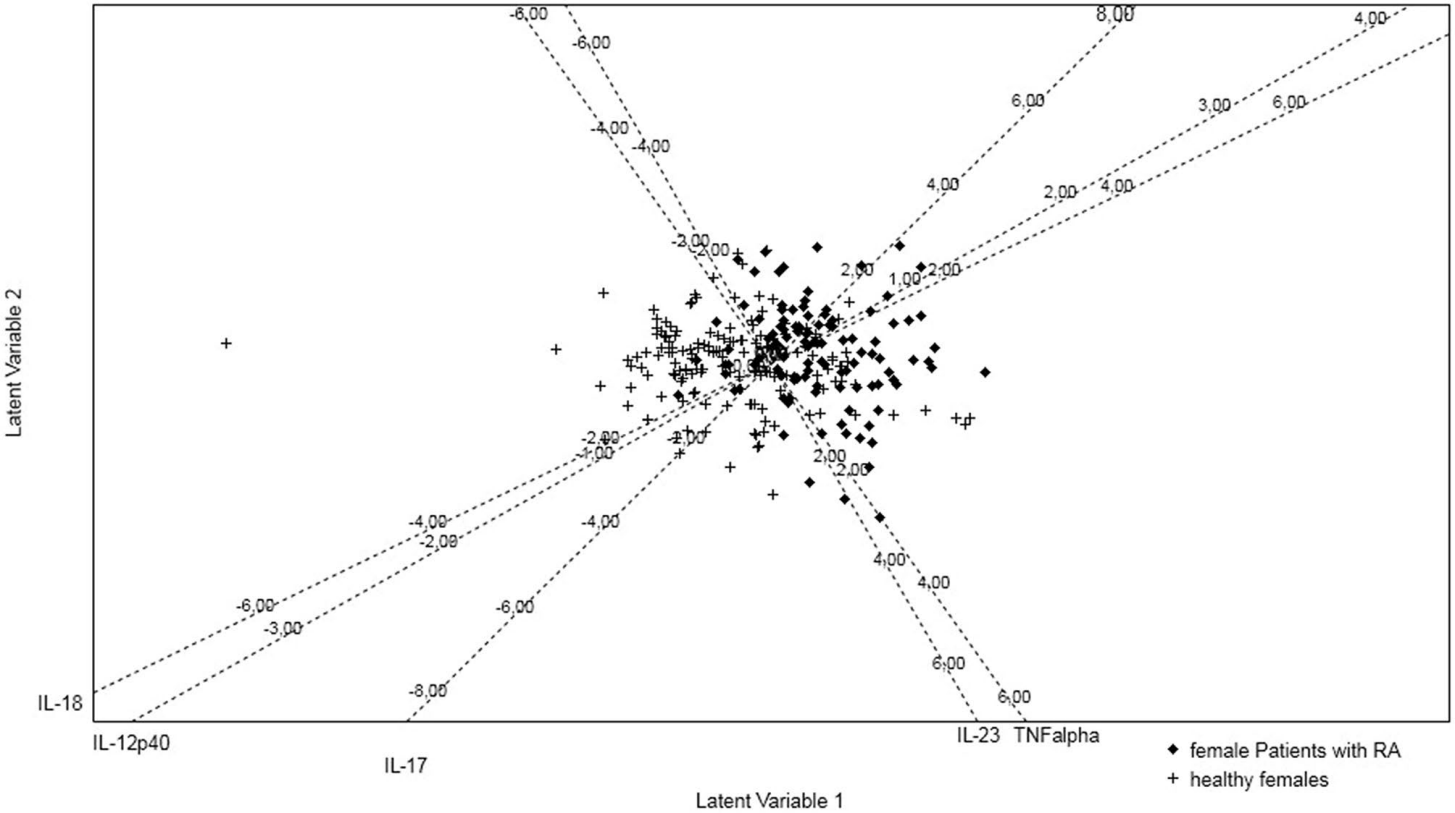
Standardized biplot generated using PLS-DA analysis visualizing the multivariate cytokines profiles of 78 female RA patients and 51 healthy controls in two-dimensional subspace based on their individual scores on Latent Variable 1 and Latent Variable 2.

The results obtained using the PLS-DA are illustrated by a standardized biplot (Fig. 2).

PLS-DA has 76% Calibration Accuracy. RA patients are represented as “rhombs” and healthy controls as “plus” on the biplot. Dashed axes represent the cytokine variables and their relation with latent variables (LV1 and LV2). LV is linear combinations of studied cytokine variables and is chosen to maximize the linear separation between healthy women and female RA patients. LV1 and LV2 had eigenvalue bigger than 1, LV1 accounted for 68%, and LV2 for 28% of the variance in the cytokine variables (R^2^X). Both groups have low to modest overlap based on their PLS-DA projections. The standardized biplot displays the good separability between RA and control females based on their multivariate cytokine profiles.

**Figure 3:**
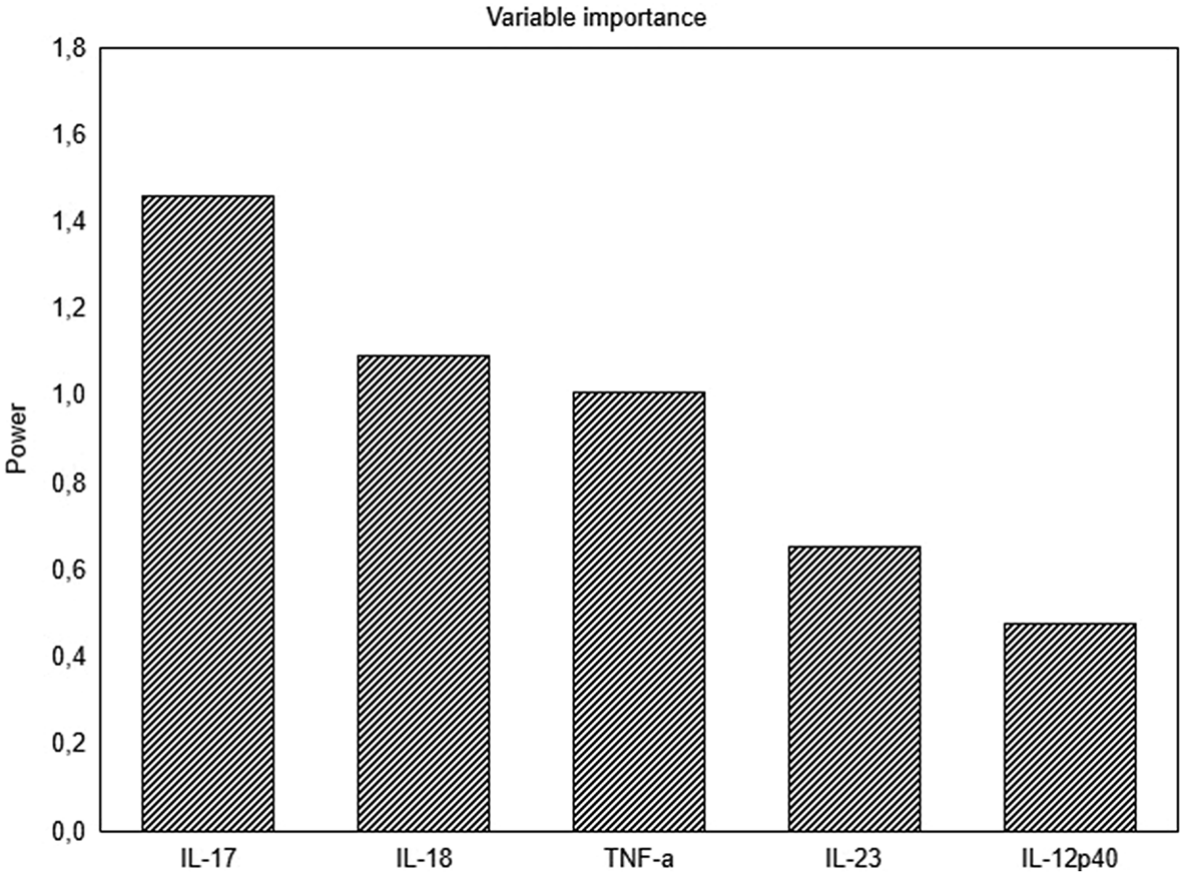
Bar graph shows which cytokines account the most for separation between groups according to PLS-DA analysis based on their VIP (Variable Importance in Projection) scores.

Furthermore, Fig. 3 presents studied cytokine variables' contribution and importance to multivariate differences between RA and healthy females according to PLS-DA.

Cytokines with the highest importance to LV are those that better discriminate between groups. Based on our results, the multivariate differences could be related mainly to IL-17A, IL-18, and TNF-α due to their biggest VIP (Variable Importance in Projection) scores, 1.5, 1.17, and 1.0, respectively. IL-6 was discarded from PLS-DA analysis since its detectability in the control cohort was less than 80%.

### Comparison of cytokine levels among female RA patients on different treatment regimens

Results comparing the cytokine serum concentration between female RA patients treated with different therapeutic regimens are presented in Table 2. No significant differences were observed between the three therapeutic subgroups regarding disease activity status, anti-CCP, and RF positivity.

**Table 2:** Serum cytokines in RA female patients` treatment subgroups.

Cytokine levels (pg/ml)	Low dose CS	csDMARDs	TCZ
IL-6	19.4 ± 25.7	21.5 ± 40.7	38.0 ± 59.1
	7.5 (3.0–28.0)	4.8 (1.4–19.4)	17.2 (9.9–43.1)
IL-17A	27.7 ± 45.9	7.5 ± 7.1	7.4 ± 11.6
	12.7 (3.0–26.1)	4.4 (2.9–9.21)	4.2 (3.1–5.6)
IL-18	269.3 ± 218.4	286.6 ± 167.0	333.7 ± 193.4
	201.4 (138.7–302.8)	241.5 (177–375.7)	322.9 (201.2–355.2)
TNF-α	6.0 ± 3.4	7.5 ± 8.8	7.1 ± 9.3
	5.2 (3.6–8.6)	6.1 (2.6–8.8)	3.6 (2.5–5.4)
IL-23	41.2 ± 73.5	23.4 ± 24.0	79.7 ± 139.5
	10.1 (8.5–24.8)	15.8 (8.8–23.8)	15.1 (5.6–129.2)
IL-12p40	121.4 ± 101.8	188.0 ± 128.6	185.0 ± 109.1
	104.4 (54.4–153.2)	172.8 (88.5–238.1)	136.1 (86.9–301.4)

Data are presented as mean ± SD and median (IQR).

*CS* corticosteroids, *csDMARDs* conventional synthetic disease modifying anti-rheumatic drugs, *IQR* interquartil range, *TCZ* Tocilizumab.

The proportion of patients with IL-6 levels above the median value was significantly higher in patients on biological therapy than other patients groups (71% vs. 40%; x^2^ = 5.89; *p* = 0.05). The highest IL-17A levels were found in subjects on symptomatic treatment with low-dose corticosteroids. The comparison of IL-17A cytokine levels among different therapeutic regimens pointed out that patients treated with IL-6R inhibitor TCZ displayed the lowest levels of IL-17A, also confirmed by the median test. Sixty-eight percent of patients on biological therapy and 41% of other patients groups demonstrated IL-17A levels below the median value (x^2^ = 6.32; *p* = 0.042). Concerning IL-18, subjects on TCZ displayed the highest levels, but no significant difference between patients’ groups according to treatment received was shown. Also, there is no significant difference in the serum TNF-α level of patients treated with TCZ and other patients groups. However, the proportion of patients with TNF-α levels below the median value was significantly higher in patients on biological therapy compared to other therapeutic groups, as demonstrated by the median test (77% vs. 43%; x^2^ = 9.56; *p* = 0.008). Concerning IL-23 levels, no significant difference was observed (*p* = 0.539; Kruskal–Wallis test), but patients on TCZ demonstrated the greatest variance in the cytokine levels. The comparison of median levels of IL-12p40 between the three therapeutic subgroups showed no significant differences.

**Figure 4:**
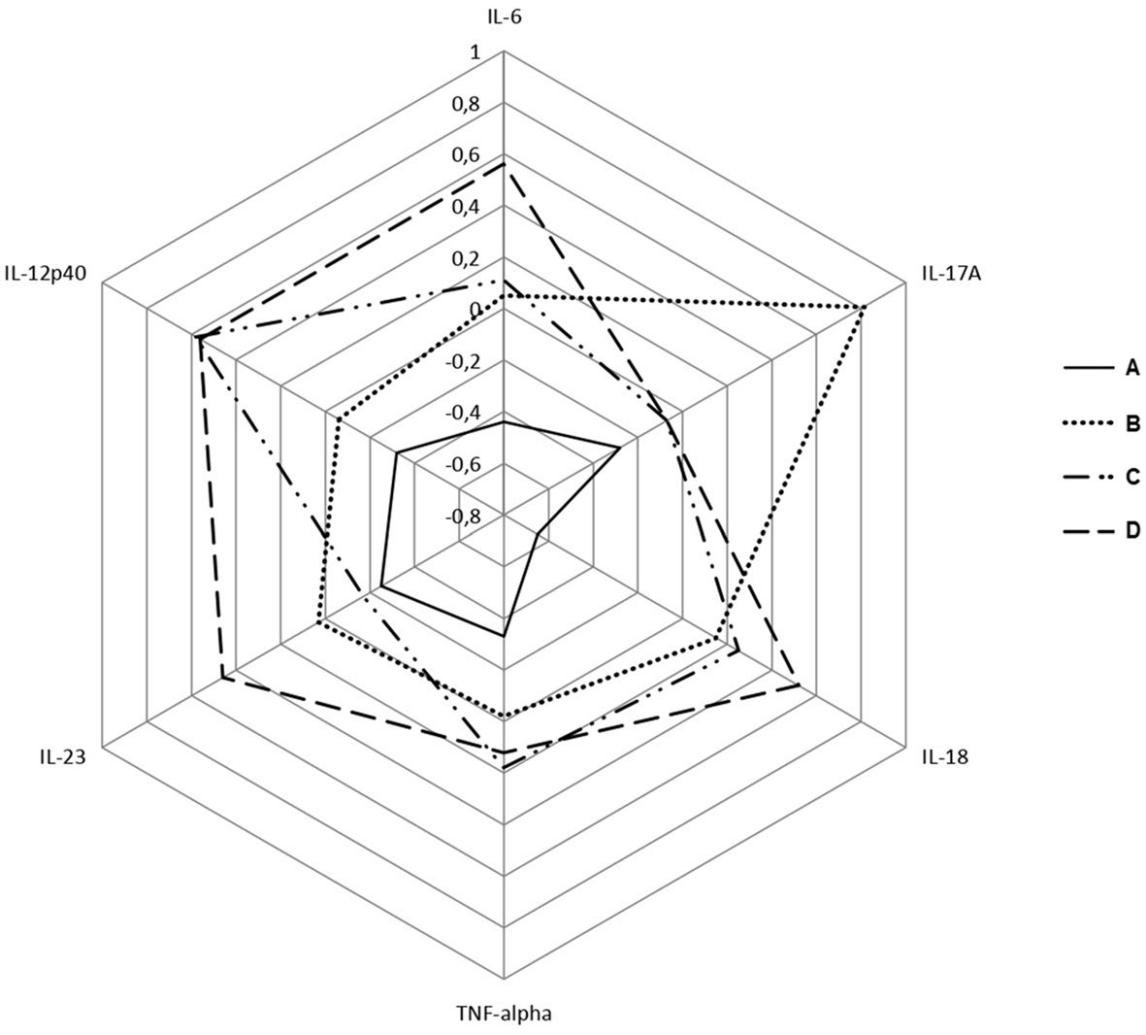
Radial chart visualizing mean cytokine levels in controls (**A**) and patients on low dose corticosteroid treatment (**B**), csDMARDs (**C**), and TCZ (**D**). Cytokine variables were normalized, mean-centered, and unit variance scaled.

An integrated picture of mean serum levels after normalization of studied cytokines in controls and patients subdivided according to their therapeutic regimes is presented in Fig. 4.

Patients on TCZ therapy showed elevated IL-6 levels and decreased IL-17A versus the rest of the patients' subgroups in the analysis. Also, the highest mean IL-23 and IL-18 in the circulation were observed among patients on TCZ.

### Serum cytokine levels in relation to RA disease characteristics

Results comparing the cytokine serum concentration between female RA patients in relation to some clinical characteristic are presented in Table 3.

**Table 3:** Significant differences in cytokine levels in relation to RA disease characteristics in female patients.

	IL-17A pg/ml	IL-6 pg/ml	TNF-α pg/ml
**Disease duration**			
< 1 years	22.8 [7.8–124.3]	4.09 [1.02–10.07]	5.9 [4.2–8.2]
> 1 years	4.4 [2.9–12.0]	11.6 [2.9—40.6]	5.0 [2.7–8.7]
	0.017*		
**Disease activity (DAS28-CRP**			
Low activity	4.8 [3.2–14.2]	1.52 [0.0–12.1]	4.3 [2.8—5.9]
Moderate activity	4.6 [3.01–13.3]	12.7 [3.3–43.4]	4.9 [ 2.9–8.04]
High activity	17.3 [4.3–24.8]	15.2 [9.4–36.7]	7.5 [ 3.1–10.6]
		0.007*	
**RF positivity**			
Negative	4.3 [2.9–6.7]	7.4 [1.8—36.4]	3.7 [ 2.5–6.3]
Positive	8.2 [ 3.4—23.2]	12.5 [3.0–34.2]	6.1 [4.0–9.1]
			0.011*

Data are presented as median (IQR).

*p-value according to Mann–Whitney U test.

Women in the early stage of RA (disease duration below 1 year) displayed significantly elevated [median and IQR] IL-17A levels than those with more prolonged disease duration: 22.8 [7.8–124.3] pg/ml vs. 4.4 [2.9–12.0]pg/ml, *p* = 0.017, Mann–Whitney U test. RA females patients with high and moderate disease activity as measured by DAS28-CRP had significantly elevated [median and IQR] IL-6 serum levels—15.2 [9.4–36.7] pg/ml for high activity group and 12.7 [3.3–43.4] pg/ml for moderate activity group as opposed to those with low activity: 1.52 [0.0–12.1] pg/ml*; p* = 0.007, Kruskal–Wallis test. Concerning the autoantibody status, RF positive RA females had significantly higher TNF-α levels in comparison to RF negative patients 6.1 [4.0–9.1] pg/ml vs. 3.7 [2.5–6.3]pg/ml, *p* = 0.011 for TNF-α, respectively, Mann–Whitney U test.

## Discussion

In the current study, we analyzed serum pro-inflammatory profiles of female RA patients and compared them with healthy women. In order to reach conclusions and to formulate promising therapeutic targets, we aimed to refine knowledge of how to rank pro-inflammatory cytokines regarding their relative importance in RA. For that reason, we applied a sophisticated analysis of discrimination and classification—PLS-DA.

In line with the previous studies*,* all pro-inflammatory cytokines assayed were significantly elevated in 'sera compared to healthy controls with IL-6 and IL-17A being dominated^12–14^. Serum profiles of pro-inflammatory cytokines in female RA patients showed the most significant fold change for IL-6, IL-17A, and IL-23. These data support the leading role of the IL-23/IL-17 immune axis in the pathogenesis of RA. Consistent with recent findings for a pivotal role of Th17 in disease initiation and progression, RA is often classified as Th17 dependent disease^15^. In RA, Th17 cells exert their pro-inflammatory and destructive effect by synthesizing several cytokines, such as IL-17A/F, IL-6, TNF-α^16^. Pro-inflammatory cytokine ambiance increases synovial neovascularization and favors the influx of immunocompetent cells into the synovium. Moreover, under pro-inflammatory cytokine background (IL-6, IL-17A/F), fibroblast-like cells, a normal component of the synovial stem cell niche, become activated, proliferate, and release matrix metalloproteinases, promote cartilage destruction and neo-antigen formation^16^. Furthermore, infiltrating monocytes become polarized into M1 macrophages or mature dendritic cells (DC). M1 macrophages and DC up-regulate co-stimulatory surface molecules, major histocompatibility complex (MHC) class II molecules, and cytokine production (TNF-α, IL-12, IL-23, IL-6). They thus can further activate naïve T effector cells or mature into destructive osteoclasts^17^. Thus elevated pro-inflammatory cytokine levels fuel the self-sustaining autocrine loop of autoimmune dysregulations in RA.

Several findings in our study support the involvement of cytokines in the pathogenesis of the disease. First, IL-17A was significantly higher in the early phase of RA in contrast to later stages of disease progression, which agrees with the notion of hierarchical dominance of different pro-inflammatory cytokines in the course of RA^18^. IL-17A is an early initiator of inflammation and dominates in the pre-clinical phase of the RA, whereas IL-17F contributes mainly to the chronic phase of joint inflammation together with TNF-α and IL-6^18,19^. In the same direction are our results for enhanced IL-6 levels in patients with longer disease duration. Second, our analysis found that IL-6 circulating levels have been linked to a higher disease activity state, suggesting the crucial inflammatory role of IL-6 in RA pathogenesis. Previous studies have also reported an association of high serum IL-6 levels with disease activity and severity^14,20,21^. Additionally, we found significantly higher TNF-α levels in RF positive RA females compared to RF negatives patients, further indicating the role of TNF-α in autoimmune dysregulation and clinical presentation of RA. A study by Takeuchi et al. has reported a correlation of RF/anti-CCP titers with TNF level^22^. Although the mechanism underlying the correlation between RF/anti-CCP and TNF levels is unknown, experimental data showed the ability of RF to amplify the anti-CCP induced production of pro-inflammatory cytokines by macrophages^23^. A “vicious cycle” has been suggested to exist in female RA patients, where RF/anti-CCP promotes TNF production, contributing to further induction of RF/anti-CCP through a still unknown mechanism^22^.

Also noteworthy are the results of Partial Least Squares-Discriminant Analysis, which aimed to identify the cytokines that have the greatest weight in distinguishing female RA patients from healthy women and, therefore, act a leading role in disease pathogenesis. According to the analysis results, IL-17A, IL-18, and TNF-α were of higher importance rank compared to IL-23 and IL-12p40. However, it should be noted that IL-6 was automatically excluded from the analysis due to its undetectable levels in part of the controls. The increased levels of IL-18 in female RA patients’ sera observed could be linked unambiguously to the involvement of the NLRP3 inflammasome^24^. Following NLRP3 inflammasome assembly, two important cytokines are released: IL-1β and IL-18, respectively, but IL-1β was not an object of our analysis^24,25^. The list of potent NLRP3 activation stimuli includes many pathogen-associated molecular patterns and endogenous stress and danger signals. In healthy individuals, NLRP3 assembly resides innate defense strategy provoked by invading pathogens^26^. Thereby, as Fig. 3 displays, healthy subjects have the lowest standardized levels of IL-18 compared to the standardized levels of the other tested cytokines, and IL-18 serum levels were significantly higher in all patients’ groups compared to controls. Recent findings highlight that NLRP3 inflammasome pathway over-activation is an integral part of the framework of RA immune dysregulation as its activation could be correlated with disease activity^27,28^. Activation of the NLRP3 pathway is well established in macrophages, but according to recent findings, Th17 cells in RA also exhibit increased NLRP3 activity, and levels of IL-1β and IL-18 correlated directly with IL-17 levels^29^.

Furthermore, NLRP3 activation in CD4+ helper cells was shown to promote Th17 differentiation. Inhibition of the IL-18 production could lead to decreased Th17 differentiation^28^. Thereby, the higher importance rank of IL-18 identified in our study could be explained by NLRP3 inflammasome over-activation in Th17 cells in RA, which is documented in other publications^28^. Therapy with biological agents targeting IL-18 in addition to the Th17 axis may be an adequate approach in female RA patients.

In addition, many previous studies have been shown that serum and synovial IL‐18 levels are correlated with disease activity in RA and their pivotal role in maintaining the joint inflammation in RA, summarized in review papers^29,30^. The newest support for IL-18 therapeutic targeting in RA came from experiments with IL-18Rα knockout (KO) mice model of induced experimental arthritis^31^. The results from this study demonstrated that inhibition of the IL-18/IL-18Rα signaling pathway inhibited not only the proliferation of autoreactive T cells but also the suppression of IL-6, IL-18, TNF, and IFN-γ serum levels. That strongly correlates with decreased bone erosion and synovitis in IL-18Rα knockout (KO) mice model with experimentally induced arthritis. Further studies on the IL-18/IL-18 receptor signaling pathway in RA are required to clarify using this therapeutic approach.

In this study, we have also tried to evaluate serum cytokine levels depending on the therapy. Herein, we have demonstrated altered cytokine production in female RA patients on different treatment regimens. Patients on TCZ therapy had the highest circulating levels of IL-6, IL-18, and IL-23 and lowest levels of IL-17A compared to other therapeutic groups and median TNF-α level similar to the healthy controls. The possible interpretation of this observation is that the free IL-6 cannot induce intracellular signals due to the occupation of IL-6R by TCZ. Blocking IL-6 mediated signaling is responsible for reducing IL-17A, and TNF-α levels in female RA patients treated with TCZ. The reasons why IL-6 synthesis is continuously induced in RA remain unknown, and even the TCZ treatment does not lead to a reduction in the intrinsic production of IL-6, as was shown in our study and also in other publications^14,32^. This might be caused by the inhibition of IL-6R-mediated clearance, as proposed by Nishimoto et al.

On the other hand, inhibition of IL-6 signaling could increase IL-6 levels through an autocrine loop in an attempt to restore the hampered IL-6 signaling. Alternatively, the higher IL-6 levels in the sera of these patients could be explained by their severe disease course and increased disease activity that subsequently required treatment with biological DMARDs. Besides, the highest level of IL-18 in sera of patients treated with Tocilizumab shows the necessity of other treatment approaches.

However, our study has some limitations. This is a case–control study, and the cytokine levels are measured at a fixed time point without considering the baseline characteristics of the disease. Also, the female RA patients recruited constitute a heterogeneous group in terms of disease duration, autoantibody status, and treatment regimens. It is also important to note that the number of patients included in the three therapeutics groups is relatively small, especially those on TCZ treatment. The lack of data on cytokine levels prior to initiating therapy may be why we do not find a reduction in studied cytokine levels in patients on biologic treatment. In this regard, findings of cytokine levels in TCZ-treated patients should be interpreted with caution.

## Conclusion

In conclusion, our data support the pivotal role of IL-18 in addition to IL-6, IL-17A, and TNF-α as the hierarchical cytokines in the pathogenesis of RA, particularly valid for women. Th17-related cytokine profile in RA was altered after TCZ treatment. Our study also emphasizes the role of IL-18 in RA as one of the continuously and profoundly dysregulated cytokines, which might be regarded as a "vulnerable node" in the cytokine network in this chronic inflammatory condition.

## Material and methods

### Study subjects

In this cross-sectional study, we prospectively included 78 female RA patients attending the Rheumatology Clinic of University Hospital “St. Ivan Rilski” in Sofia. Subjects meeting the ACR/EULAR 2010 RA Classification Criteria and age ≥ 18 years were termed as eligible for further admission in our study^33^. Patients with a history of other inflammatory rheumatic or autoimmune disorders, malignancy, significant unstable or uncontrolled acute or chronic disease were excluded from the analysis. RA patients enrolled consisted of 78 women (mean age ± SD, 45.2 ± 13.7 years). The mean (± SD) disease duration was 9.3 ± 8.5 years. Serum levels of C-reactive protein (CRP), rheumatoid factor (RF), and anti-cyclic citrullinated peptide antibodies (anti-CCP) were measured. 73% of the patients were RF positive and 59% anti-CCP positive. Disease activity was assessed by the 28-joint Disease Activity Score calculated using CRP level (DAS28-CRP) and by Patient Global Assessment (PGA) using 100 mm visual analogous scale (VAS). Each patient's functional ability level was assessed using the Health Assessment Questionnaire Disability Index (HAQ-DI)^34,35^. Further on, 50% of subjects enrolled were treated with conventional synthetic DMARDs (csDMARDs) (Methotrexate, Leflunomide, or Sulfasalazine), 14.1% received IL-6R inhibitor Tocilizumab (TCZ), and 35.9% were on therapy with a low dose of systemic corticoids (up to 10 mg/day) due to contraindications or intolerance to DMARDs at the time of blood collection. Demographic and clinical parameters of study subjects are summarized in Table 4.

**Table 4:** Demographic characteristics and clinical data of female RA patients and healthy women.

	RA	Healthy women
*n*	78	51
Age (years)	45.2 ± 13.7	43.04 ± 13.5
Disease duration (years)	9.3 ± 8.5	
Patients with + RF (%)	57 (73)	
Patients with + anti-CCP (%)	46 (59)	
CRP (mg/l)	26.7 ± 50.4	
DAS28-CRP	4.73 ± 1.4	
PGA (mm)	53.2 ± 22.2	
HAQ-DI	1.3 ± 0.7	
Therapy		
CS (%)	28 (35.9)	
csDMARDs (%)	39 (50.0)	
TCZ (%)	11 (14.1)	

Data are presented as mean ± SD.

*Anti-CCP* anti-cyclic citrullinated peptide antibody, *CS* corticosteroids, *csDMARDs* conventional synthetic DMARD, *CRP* C reactive protein, *DAS28-CRP* Disease Activity Score 28 calculated using C-reactive protein level, *HAQ-DI* Health Assessment Questionnaire Disability Index, *PGA* Patient Global Assessment (range: 0–100 mm), *RA* Rheumatoid arthritis, *RF* rheumatoid factor, *SD* standard deviation, *TZC* Tocilizumab.

Comparisons were made with 51 healthy women (mean age ± SD, 43.04 ± 13.5 years; range 20–71 years) who were consecutively recruited from a health checkup program during the study period. The aforementioned exclusion criteria were applied to the control group. The cases and controls were ethnically matched (Caucasian origin).

This study was held under the approval of the institutional ethics committee at University Hospital “St. Ivan Rilski”-Sofia, Bulgaria (Decision number 6, 29 November 2016), and all subjects voluntarily signed informed consent in compliance with the ethical standards of the Helsinki Declaration of 1964 and its further amendments ^36^.

### Blood samples

Blood samples were collected from all participants in gel/clot activator vacutainer tubes. The blood samples were allowed to clot at room temperature for 30 min before centrifugation. Serum samples were removed and frozen in small aliquots at − 70 °C until the available enzyme-linked immunosorbent assay (ELISA) analysis.

### Quantification of serum cytokine concentrations.

According to the manufacturer's instructions, serum levels of IL-6, IL-12p40, IL-17A, IL-18, IL-23, and TNF-α were quantified by commercially ELISA kits. ELISA kits for IL-6, IL-12p40, IL-23, and TNF-α were purchased from Invitrogen Corporation (Camarillo, CA, USA) and IL-17A, IL-18 from eBioscience (Vienna, Austria). A standard curve constructed with the kit’s standards was used to determine the cytokine concentration expressed in picograms per ml (pg/ml). Serum samples of patients and controls were run in duplicate and analyzed together in the same analytic batch. The minimum detection levels were less than 2.0 pg/ml for IL-6 and IL-12p40, 0.05 pg/ml for IL-17A, 9 pg/ml for IL-18, 4 pg/ml for IL-23, and 0.09 pg/ml for TNF-α. Values below the detection limits were set as zero.

### Data analyses

The normality of studied cytokines distributions was assessed using Kolmogorov–Smirnov and Shapiro–Wilk tests. For multivariate analysis purposes, cytokine variables were transformed using inverse density function and were mean-centered and variance scaled. To examine univariate differences in cytokine levels between female RA patients and healthy women, Student's Test, and Man-Whitney U test were used where appropriate. Adjustment due to multiple hypotheses testing was applied in compliance with the Benjamini and Hochberg method, and q values were estimated. To visualize the multivariate differences between cytokine profiles of female RA patients and healthy controls, Partial Least Squares-Discriminant Analysis (PLS-DA) was performed. PLS-DA is a supervised dimensionality reduction technique that finds the best separation between both groups. It “projects” multidimensional cytokine data into lower-dimensional subspace, thus producing new variables termed latent variable (LV). The results from the analysis are visualized using a standardized bi-plot and bar graph presenting cytokines contribution or importance for distinguishing between healthy women and female RA patients. Spearman’s rank correlation test was performed to investigate the correlations between cytokine levels within female RA patients. Furthermore, to examine the differences in cytokine levels between female RA patients on different therapeutic regimens and healthy women, one-way ANOVA analysis or Kruskal–Wallis with corrections for multiple testing and median test were used.

### Ethics approval

This study was held under the approval of the institutional ethics committee at University Hospital “St. Ivan Rilski”-Sofia, Bulgaria (Decision number 6, 29 November 2016).

### Consent to participate

Informed consent was obtained from all subjects enrolled in the study.

## Data Availability

The datasets generated during and/or analysed during the current study are available from the corresponding author on reasonable request.
